# Prevalence of bovine mastitis and antibiotic susceptibility patterns of bacterial isolates in the Jaffna District, Sri Lanka

**DOI:** 10.3389/fvets.2026.1908298

**Published:** 2026-08-26

**Authors:** Kalaivizhi Varathanathan, Croos Antony Niroshan Rajeev, Susantha Piratheepan, Thasanthan Loganathan

**Affiliations:** 1 Department of Animal Science, Faculty of Agriculture, Eastern University of Sri Lanka, Chenkaladi, Sri Lanka; 2 Department of Animal Science, Faculty of Agriculture, University of Jaffna, Kilinochchi, Sri Lanka; 3 Department of Radiography/Radiotherapy, Faculty of Allied Health Sciences, University of Peradeniya, Peradeniya, Sri Lanka

**Keywords:** antibiotic susceptibility, antimicrobial resistance, bovine mastitis, California Mastitis Test, Jaffna, risk factors, Sri Lanka, *Staphylococcus aureus*

## Abstract

**Background:**

Bovine mastitis is one of the most economically important infectious diseases affecting the global dairy industry. In the Jaffna district of Northern Sri Lanka, information on mastitis prevalence, bacterial aetiology, and antimicrobial resistance remains limited, constraining evidence-based disease management.

**Objectives:**

To determine the California Mastitis Test (CMT)-based prevalence of bovine mastitis in the Jaffna district, identify causative bacterial pathogens, evaluate antibiotic susceptibility patterns, and assess farm-level risk factors associated with mastitis occurrence.

**Methods:**

A cross-sectional study was conducted from April to September 2024 across all 15 veterinary ranges of Jaffna district. A total of 200 milk samples from lactating dairy cattle were screened using the CMT. Positive samples were cultured, and bacterial isolates were identified through Gram staining and standard biochemical tests. Antibiotic susceptibility testing was performed using the Kirby-Bauer disk diffusion method against eight antibiotics following CLSI (2021) guidelines, and Multiple Antibiotic Resistance (MAR) indices were calculated. A structured questionnaire administered to 200 farmers collected data on management, breed, hygiene, and milking practices. Associations between risk factors and mastitis occurrence were analysed using chi-square tests and binary logistic regression.

**Results:**

The overall prevalence of bovine mastitis was 10.0% (20/200), with all CMT-positive samples yielding bacterial growth. *Staphylococcus aureus* was the predominant isolate (50%), followed by *Streptococcus agalactiae* (30%), *Escherichia coli* (10%), and *Klebsiella* spp. (10%). Enrofloxacin showed the highest susceptibility (90%; MAR = 0.14), followed by Doxycycline (60%) and Ciprofloxacin (55%), whereas Oxytetracycline exhibited the lowest efficacy (15%; MAR = 0.45). Semi-intensive farming (aOR = 1.72; *p* = 0.05), European crossbred cattle (aOR = 3.12; *p* < 0.05), milk yield >10 L/day (aOR = 2.45; *p* = 0.036), absence of post-milking teat dipping (aOR = 2.67; *p* < 0.05), and non-use of gloves during milking (aOR = 2.90; *p* < 0.05) were significant risk factors.

**Conclusion:**

Bovine mastitis remains a significant challenge in Jaffna district, with *S. aureus* as the dominant pathogen. Enrofloxacin was the most effective antibiotic, while high resistance to Oxytetracycline highlights concerns regarding antimicrobial resistance. Improved milking hygiene and antibiogram-guided treatment strategies are recommended.

## 1 Introduction

Mastitis, the inflammation of the mammary gland, is globally recognised as the costliest infectious disease of dairy production, imposing multi-dimensional losses: reduced milk yield (100–500 kg per cow per lactation), deteriorated milk quality, elevated somatic cell counts (SCC), increased veterinary expenditure, premature culling of productive cows, and, in severe cases, permanent udder damage (1, 2). Beyond farm-level economics, mastitis carries public-health implications, since mastitic milk may harbour zoonotic pathogens and antibiotic residues that compromise food safety and accelerate the global antimicrobial resistance (AMR) crisis (3, 4).

Sri Lanka’s livestock sector supports approximately 1.11 million cattle and 0.31 million buffaloes nationally (5). The Jaffna district of the Northern Province maintains a registered dairy cattle population of 73,168 head across 15 veterinary ranges, predominantly comprising Cross European breeds (57%), local breeds (22%), and Cross Indian breeds (21%) (6). Smallholder dairy farming, largely semi-intensive in character, constitutes a critical livelihood for farming families in this post-conflict region. However, fragmented landholdings, limited veterinary infrastructure, and historically poor access to extension services render these herds particularly vulnerable to endemic mastitis (7).

Nationally, mastitis was the most frequently reported livestock disease in Sri Lanka in 2012, with 11,264 cases documented (975 in the Northern Province) and an estimated annual economic loss of SLR 4.3 million (8). The primary causative agents are classified as contagious (*Staphylococcus aureus, Streptococcus agalactiae, Mycoplasma bovis*) or environmental (*Escherichia coli, Klebsiella* spp.*, Streptococcus uberis*), each requiring distinct management strategies (9, 10). Rational antibiotic therapy depends on local antibiogram data, which remain virtually absent for the Jaffna district in the peer-reviewed literature.

The California Mastitis Test (CMT) is the most widely validated, field-deployable screening tool for subclinical mastitis, detecting elevated cellular nuclear protein in milk as a proxy for SCC (11, 12). Integration of CMT screening with laboratory pathogen identification and antibiotic susceptibility testing (ABST) provides the evidence base for antimicrobial stewardship and resistance surveillance in dairy herds (13).

Despite the recognised significance of mastitis in Northern Sri Lanka, no peer-reviewed study has systematically characterised its prevalence, microbial aetiology, antibiotic susceptibility profile, and farm-level risk factors across the Jaffna district. This study addresses that evidence gap through a cross-sectional design spanning all 15 veterinary ranges, with the objectives of: (i) determining CMT-based mastitis prevalence; (ii) identifying causative pathogens by cultural and biochemical methods; (iii) characterising antibiotic susceptibility against eight antibiotics by disk diffusion; and (iv) identifying modifiable farm-level risk factors through a structured questionnaire survey and logistic regression.

## 2 Materials and methods

### 2.1 Study area, design, and ethical considerations

A cross-sectional observational study was conducted from April to September 2024 in the Jaffna district (09°40′N, 80°10′E), Northern Province of Sri Lanka. The district spans approximately 1,025 km^2^ and is organised into 15 Divisional Secretary divisions, each served by a dedicated veterinary range. The total registered dairy cattle population is 73,168, comprising Cross European breeds (41,454; 57%), local breeds (16,568; 22%), and Cross Indian breeds (15,146; 21%) (6). All sampling was performed by licensed veterinary officers; owner informed consent was obtained for all farms. Sample collection employed standard minimally invasive aseptic procedures consistent with institutional animal-care guidelines.

### 2.2 Sample size calculation and sampling strategy

Sample size was calculated using the formula *n* = Z^2^P(1 − P)/d^2^, where Z = 1.96 (95% CI), P = expected prevalence of 15% (based on (8)), and d = 0.05 (precision), yielding a minimum of 196, rounded to 200. Stratified random sampling proportional to herd size was applied across all 15 veterinary ranges. Milk samples were collected from individual lactating cows presenting to the Veterinary Investigation Centre (VIC), Jaffna, through field visits to registered farms, and from farmers at Yarlco milk collection centres. Inclusion criteria were: currently lactating, no antibiotic treatment within 14 days, and owner consent.

### 2.3 Questionnaire survey and risk factor data collection

A pretested structured questionnaire was administered to 200 dairy farm owners by trained enumerators via direct interview. Variables captured included: farming system type (extensive/semi-intensive/intensive), breed, lactation stage, daily milk yield, milking method (hand/machine), milking equipment type and sterilisation practice, water source, post-milking teat-dipping compliance, use of gloves during milking, parturition management (communal versus separate calving pen), mastitis treatment history, and animal isolation practices. Data reliability was cross-validated against VIC records.

### 2.4 California Mastitis Test (CMT)

All 200 milk samples were screened using the California Mastitis Test (CMT; Vet-Lab Supply, New Zealand). Foremilk (2–3 squirts) was discarded; approximately 2 mL of representative quarter milk was collected into the CMT paddle under aseptic conditions. Equal volumes of CMT reagent (3% sodium lauryl sulphate, w/v) were added and swirled gently for 10–15 s. Results were graded semi-quantitatively: Negative (−), homogeneous liquid; Trace (+/−), transient gel formation; Weak positive (+), distinct gel dissolving with agitation; Distinct positive (++), immediate gel formation, thickened; Strong positive (+++), complete gel with central peak. Samples scoring (+) or above were classified as CMT-positive and subjected to bacteriological investigation. Somatic cell count (SCC) equivalents were estimated from CMT scores using the validated conversion: Trace ≈ 300,000–500,000 cells/mL; + ≈ 400,000–1,500,000; ++ ≈ 800,000–5,000,000; +++ ≥ 5,000,000 cells/mL (12).

### 2.5 Sample collection and bacteriological culture

Teat orifices were disinfected with 70% isopropyl alcohol–soaked swabs prior to sampling. Milk samples (10 mL) were collected aseptically into sterile 20 mL screw-cap bottles and transported on ice to the laboratory within 2 h of collection. Each CMT-positive sample was inoculated in triplicate by sterile loop onto: Nutrient Agar (37 °C, 24–48 h); MacConkey Agar (37 °C, 24–48 h, selective for Gram-negative organisms); Eosin Methylene Blue (EMB) Agar (37 °C, 24–48 h, differential for lactose fermenters); and 5% Sheep Blood Agar (37 °C, 24 h) to assess haemolysis. All inoculations were performed in a laminar flow safety cabinet under a Bunsen burner flame. Morphologically distinct colonies were sub-cultured on Nutrient Agar for pure-culture isolation.

### 2.6 Pathogen identification

#### 2.6.1 Gram staining

Heat-fixed smears were prepared from pure colonies on clean, grease-free glass slides. Sequential treatment with crystal violet (60 s), Gram’s iodine mordant (60 s), acetone–ethanol decoloriser (50:50 v/v; 5–10 s), and safranin counterstain (60 s) was performed with sterile-water rinses between steps. Smears were examined under oil immersion (100×) using a light microscope (Olympus CX23). Violet-stained organisms were classified as Gram-positive; pink-stained organisms as Gram-negative.

#### 2.6.2 Presumptive biochemical tests

Catalase test: a loopful of Gram-positive colony was added to 3% H₂O₂ on a glass slide; evolution of O₂ bubbles within 10 s indicated catalase-positive organisms (Staphylococcus spp.), while absence indicated catalase-negative cocci (*Streptococcus* spp.). Oxidase test: Gram-negative isolates were tested using HiMedia oxidase strips; colour development within 10 s indicated cytochrome-c oxidase positivity.

#### 2.6.3 Confirmatory biochemical panel for Gram-negative organisms

Standard biochemical test batteries were performed: Triple Sugar Iron (TSI) agar (acid/acid, gas, H₂S); Sulphide-Indole-Motility (SIM) medium (H₂S, indole by Kovac’s reagent, motility); Christensen’s Urease agar; and Simmons’ Citrate agar. Results were interpreted at 24–48 h at 37 °C per Bergey’s Manual. Differential profiles were as follows: *E. coli* (TSI A/A, gas+, H₂S−; indole+; citrate−; urease−; motile/non-motile) versus *Klebsiella* spp. (TSI A/A, gas+, H₂S−; indole−; citrate+; urease+; non-motile; encapsulated on Gram stain).

### 2.7 Antibiotic susceptibility testing (ABST) — Kirby-Bauer disk diffusion

ABST was performed per CLSI M02 (2021) guidelines on Mueller Hinton Agar (MHA; Oxoid, UK). Bacterial suspensions were prepared in 0.9% NaCl and adjusted to 0.5 McFarland turbidity standard (~1.5 × 10^8^ CFU/mL) using a DensiMeter (HiMedia). Suspensions were swabbed uniformly across MHA surfaces with sterile cotton swabs; plates were dried for 5 min before disc application. Antibiotic discs (HiMedia Laboratories, India) were placed ≥24 mm apart: Enrofloxacin (EX, 5 μg), Doxycycline (DO, 30 μg), Ciprofloxacin (CIP, 5 μg), Cephalexin (CN, 30 μg), Trimethoprim-sulfamethoxazole (SXT, 25 μg), Gentamicin (GN, 10 μg), Erythromycin (E, 15 μg), and Oxytetracycline (OTC, 30 μg). Plates were incubated at 37 °C for 18–24 h; inhibition-zone diameters were measured to the nearest mm under bright oblique light. Isolates were classified as Susceptible (S), Intermediate (I), or Resistant (R) per CLSI breakpoints for veterinary pathogens. The Multiple Antibiotic Resistance (MAR) index was calculated as: MAR index = number of antibiotics to which the isolate was resistant / total antibiotics tested. An MAR index > 0.2 indicates a high-risk contamination source (14).

### 2.8 Statistical analysis

Data were managed in Microsoft Excel 2019 and analysed using IBM SPSS Statistics v23.0. Mastitis prevalence was calculated as (positive/total) × 100, with exact 95% confidence intervals (Wilson method). Pearson’s chi-square (χ^2^) test assessed associations between categorical risk factors and mastitis occurrence; for pathogen groups with expected cell counts < 5 (*E. coli, Klebsiella* spp.), Fisher’s exact test was used instead. Binary logistic regression with forward stepwise selection (Wald criterion) identified independent predictors of mastitis occurrence; results are reported as adjusted odds ratios (aOR) with 95% confidence intervals (CI). Inhibition-zone diameters were compared across pathogen groups using one-way ANOVA with Tukey’s honestly significant difference (HSD) post-hoc test. The Multiple Antibiotic Resistance (MAR) index was calculated for each isolate as described in Section 2.7. A *p*-value < 0.05 was considered statistically significant throughout.

## 3 Results

### 3.1 CMT-based mastitis prevalence and bacterial isolation rate

Of 200 individual milk samples screened, 20 (10.0%; 95% CI: 6.6–14.9%) tested CMT-positive, yielding an overall mastitis prevalence of 10.0% in the Jaffna district during the April–September 2024 study period (Table 1). All 20 CMT-positive samples yielded bacterial growth on primary culture, producing a 100% culture-positivity rate, consistent with prior cross-sectional studies in tropical smallholder dairy systems reporting culture-confirmed mastitis in 8–15% of milk samples (15, 16). Questionnaire data revealed a higher historical prevalence: 26.5% (36/136) of surveyed farmers reported at least one episode of mastitis in their herd over the preceding 12 months, suggesting that the point prevalence measured by CMT substantially underestimates true herd-level disease burden through subclinical infections (3). The VIC Jaffna recorded 400 CMT-positive cases in full-year 2024, of which 200 occurred during the six-month study window, reflecting the endemic, year-round nature of mastitis in the region. All 50 bulk milk samples from Yarlco milk collecting centres were CMT-negative, consistent with the dilution effect of bulk-tank collection and the quality-gatekeeping practices enforced by collecting-centre officers — an observation corroborated by Madut et al. (17) in a comparable institutional collection setting.

**Table 1:** CMT screening outcomes and prevalence of bovine mastitis, Jaffna district, 2024.

Parameter	Samples (*n*)	Positive (*n*)	Prevalence (%)
Individual milk samples screened	200	20	10.0
Culture positivity (CMT + samples)	20	20	100.0
Bulk milk samples (collecting centres)	50	0	0.0
Farms with mastitis history (questionnaire; *n* = 136)	136	36	26.5
Farms with animals showing clinical signs	20	20	—

### 3.2 Identification of causative bacterial pathogens

Gram staining of primary isolates categorised 15 samples (75%) as Gram-positive and 5 samples (25%) as Gram-negative. Catalase testing differentiated Gram-positive cocci into Staphylococcus spp. (catalase-positive; 10 isolates, 50% of total) and *Streptococcus* spp. (catalase-negative; 6 isolates, 30%). Beta-haemolysis on blood agar and colony morphology were used for presumptive species-level differentiation. Among Gram-negative isolates, biochemical profiling (Table 2) confirmed *E. coli* (*n* = 2; 10%) and *Klebsiella* spp. (*n* = 2; 10%). Two additional Gram-negative isolates with positive oxidase reactions and non-fermentative growth patterns were provisionally classified as Pseudomonas spp. pending further speciation and were excluded from the antibiotic susceptibility analysis. The final pathogen distribution is presented in Table 3.

**Table 2:** Biochemical identification profiles of Gram-negative mastitis isolates, Jaffna District, 2024.

Isolate	Gram stain	TSI slant	TSI butt	Gas	H₂S	Indole	Citrate	Urease	Motility
*E. coli*	−ve rods	A/Y	A/Y	+	−	+	−	−	+/−
*Klebsiella* spp.	−ve encaps. Rods	A/Y	A/Y	+	−	−	+	+	−

A/Y, Acid (yellow); +, positive; −, negative; encaps., encapsulated. Identification based on CLSI M35-A2 and Bergey’s Manual of Determinative Bacteriology.

**Table 3:** Bacterial pathogens isolated from CMT-positive bovine mastitis milk samples, Jaffna district, 2024.

No.	Pathogen	Isolates (*n*)	% of positive	Gram reaction	Catalase
1	*Staphylococcus aureus*	10	50.0	+ve cocci	+
2	*Streptococcus agalactiae*	6	30.0	+ve cocci	−
3	*Escherichia coli*	2	10.0	−ve rods	−
4	*Klebsiella* spp.	2	10.0	−ve rods	−
	Total	20	100.0		

### 3.3 Antibiotic susceptibility testing (ABST)

**Figure 1:**
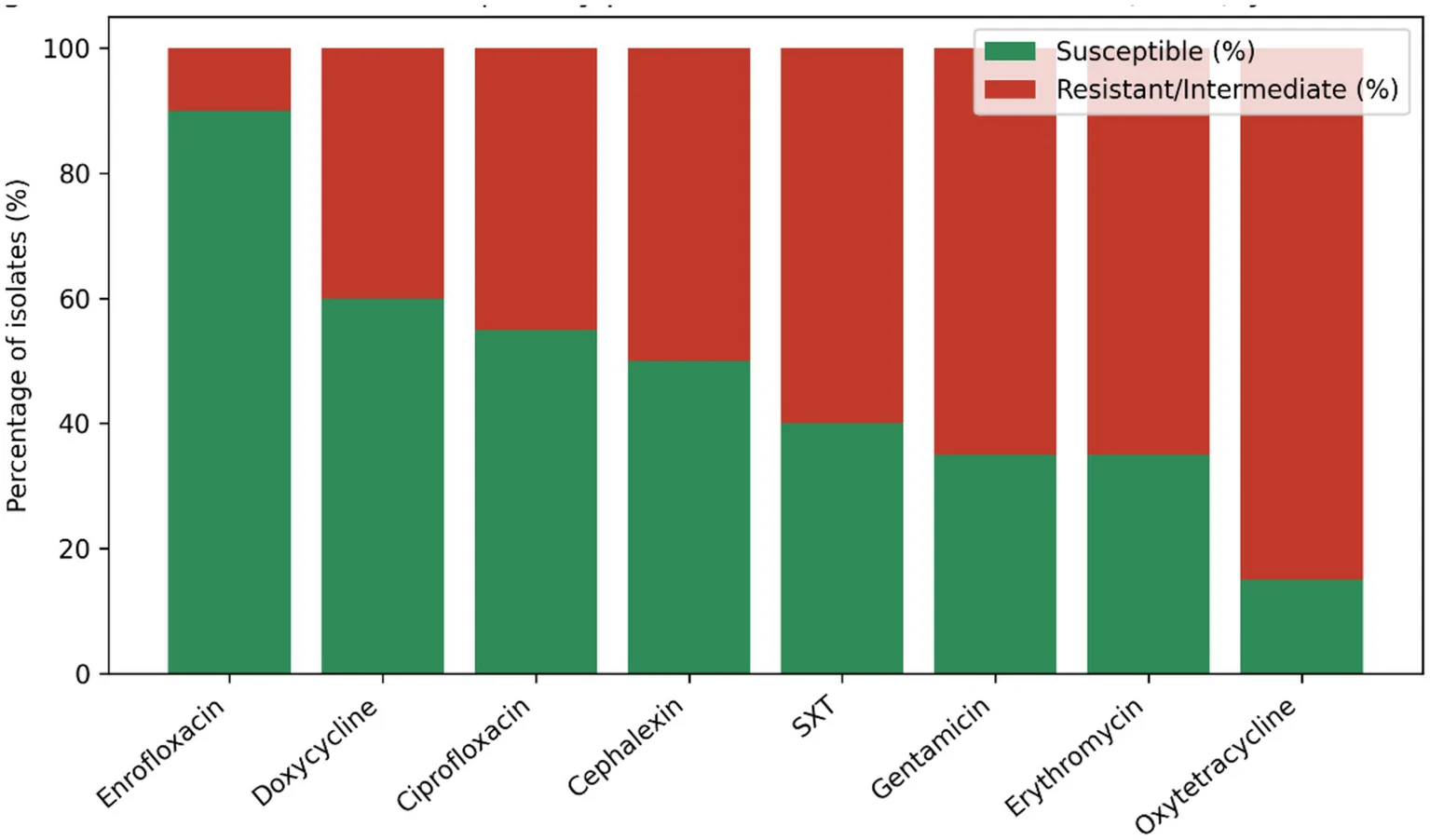
Overall antibiotic susceptibility and resistance/intermediate profile of bovine mastitis isolates (*n* = 20), Jaffna District, 2024. Green segments indicate the proportion of isolates classified as susceptible; red segments indicate intermediate or resistant isolates, by CLSI (37) breakpoints.

Inhibition-zone diameters and CLSI-interpreted susceptibility categories for all 20 isolates against eight antibiotics are summarised in Table 4. Overall susceptibility ranged from 90% (Enrofloxacin) to 15% (Oxytetracycline) a six-fold gradient (Figure 1).

**Table 4:** Antibiotic susceptibility (S), intermediate (I), and resistance (R) profiles of bovine mastitis isolates by pathogen group, Jaffna district, 2024.

Antibiotic	*S. aureus* S/I/R (*n* = 10)	*S. agalactiae* S/I/R (*n* = 6)	*E. coli* S/I/R (*n* = 2)	*Klebsiella* S/I/R (*n* = 2)	Total S (n/20)	Overall S%	MAR
Enrofloxacin	9/0/1	4/0/2	2/0/0	3/0/0	18	90%	0.14
Doxycycline	7/1/2	2/1/3	0/1/1	1/0/1	12	60%	0.22
Ciprofloxacin	5/2/3	1/1/4	0/0/2	1/0/1	11	55%	0.25
Cephalexin	6/1/3	3/1/2	0/0/2	1/0/1	10	50%	0.26
SXT	4/2/4	2/1/3	0/0/2	0/0/2	8	40%	0.32
Gentamicin	4/3/3	2/1/3	1/0/1	1/0/1	7	35%	0.34
Erythromycin	3/3/4	2/1/3	0/0/2	1/1/0	7	35%	0.36
Oxytetracycline	1/1/8	1/0/5	1/0/1	1/0/1	3	15%	0.45

S, Susceptible; I, Intermediate; R, Resistant (CLSI M02, 2021). SXT, Trimethoprim-sulfamethoxazole. MAR, Multiple Antibiotic Resistance index (>0.2 indicates high-risk source; (14)).

One-way ANOVA of inhibition-zone diameters (Table 5) showed a significant overall effect of antibiotic on zone size (*F* (7,72) = 8.94, *p* < 0.001). Tukey’s post-hoc comparisons confirmed that Enrofloxacin produced significantly larger zones (28.4 ± 6.2 mm) than Oxytetracycline (13.6 ± 3.9 mm; *p* < 0.001) and Gentamicin (16.2 ± 2.9 mm; *p* < 0.01), while differences among Doxycycline, Ciprofloxacin, and Cephalexin (22.1–20.5 mm) were not statistically significant (*p* > 0.05).

**Table 5:** Mean inhibition zone diameters (mm ± SD) of bovine mastitis isolates by antibiotic, Jaffna district, 2024.

Antibiotic	*S. aureus* (mm)	*S. agalactiae* (mm)	*E. coli* (mm)	*Klebsiella* (mm)	Overall (mm)
Enrofloxacin	28.3 ± 7.1	19.5 ± 3.2	34.0 ± 7.1	20.5 ± 1.5	28.4 ± 6.2
Doxycycline	23.6 ± 6.8	15.2 ± 4.1	—	—	22.1 ± 5.9
Ciprofloxacin	20.8 ± 9.8	12.4 ± 5.2	—	15.0 ± 0	18.6 ± 8.3
Cephalexin	20.7 ± 0.8	20.2 ± 0.4	—	20.0 ± 0	20.5 ± 0.7
Gentamicin	14.8 ± 2.0	15.5 ± 3.5	19.0 ± 0	20.0 ± 0	16.2 ± 2.9
Erythromycin	17.0 ± 4.0	21.0 ± 1.0	—	23.0 ± 0	19.2 ± 3.8
Oxytetracycline	12.5 ± 5.5	10.0 ± 0	17.0 ± 0	15.0 ± 0	13.6 ± 3.9

Values represent mean ± SD of inhibition zones from susceptible isolates only. — = no susceptible isolates in this group for this antibiotic. Overall ANOVA: F (7, 72) = 8.94, *p* < 0.001.

**Figure 2:**
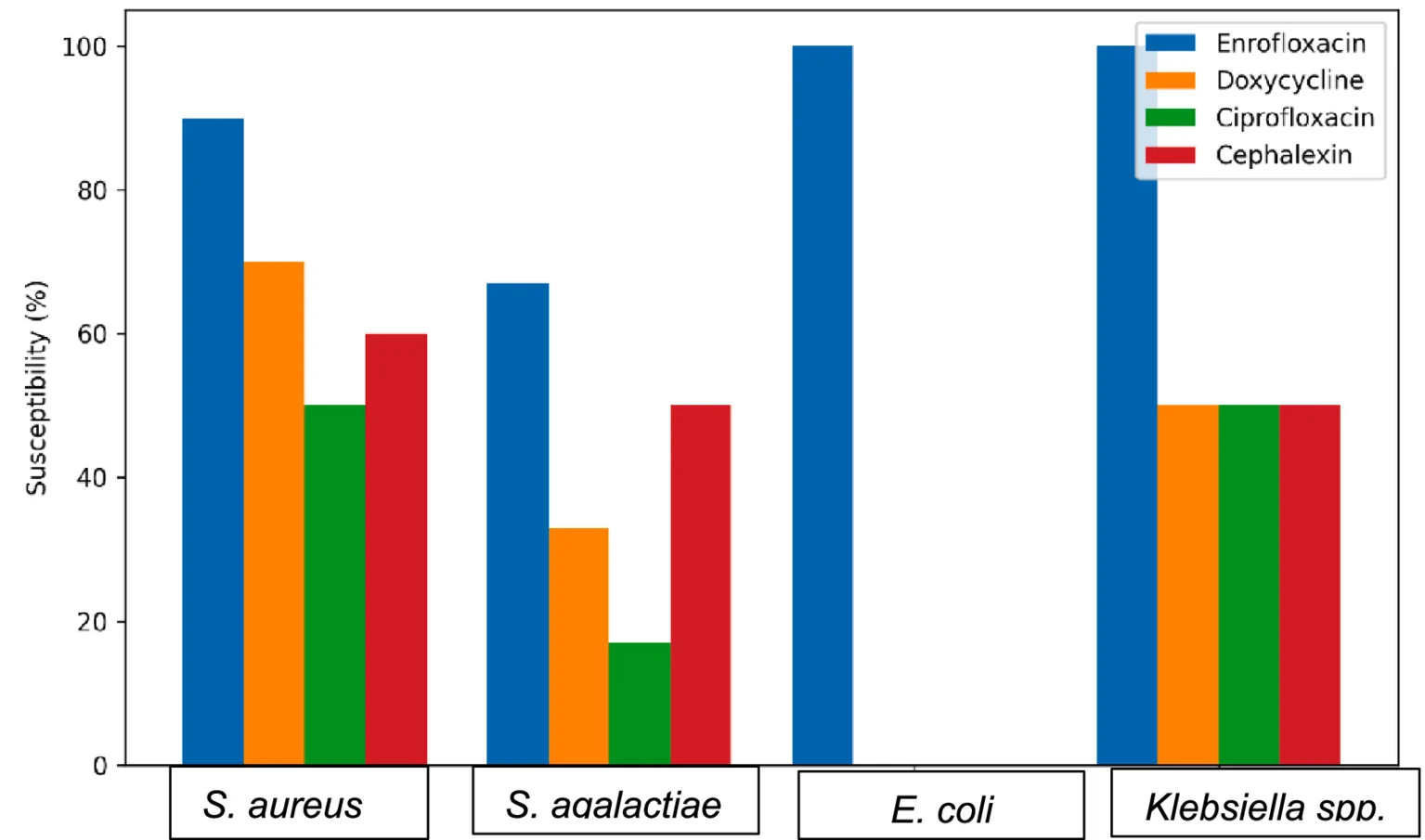
Pathogen-stratified susceptibility (%) of the four major bovine mastitis pathogens to the four most effective antibiotics (Enrofloxacin, Doxycycline, Ciprofloxacin, Cephalexin), Jaffna district, 2024.

Figure 2 illustrates the pathogen-stratified susceptibility pattern for the four most effective antibiotics.

### 3.4 Risk factor analysis

**Figure 3:**
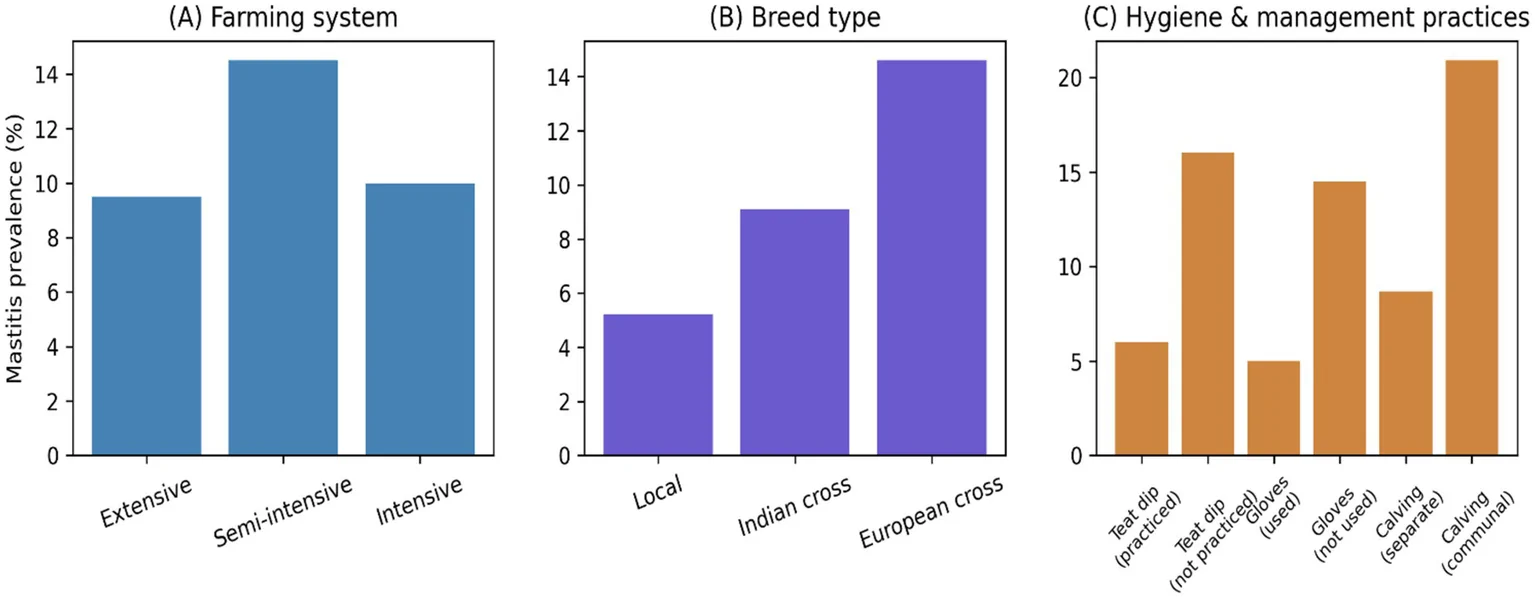
Mastitis prevalence (%) stratified by **(A)** farming system, **(B)** breed type, and **(C)** hygiene and management practices, Jaffna district, 2024.

Chi-square and binary logistic regression results for risk factors associated with mastitis prevalence are summarized in Table 6. Figure 3 presents the prevalence stratified by farming system, breed type, and hygiene practices.

**Table 6:** Risk factors associated with bovine mastitis prevalence in Jaffna district (χ^2^ analysis; *n* = 200 cows/136 farms).

Variable/Category	*n* (%)	Mastitis prev. (%)	χ^2^/*p-*value	aOR (95% CI)
Farming system			*p* = 0.05	
Extensive	14 (10.3%)	9.5		Reference
Semi-intensive	83 (61.0%)	14.5		1.72 (1.02–2.90)
Intensive	3 (2.6%)	10.0		1.05 (0.40–2.78)
Breed			*p* < 0.05	
Local breeds	16,568 (22%)	5.2		Reference
Indian cross	15,146 (21%)	9.1		1.84 (0.92–3.68)
European cross (Jersey)	41,454 (57%)	14.6		3.12 (1.67–5.83)
Milk yield > 10 L/day	99% (high)	—	*p* = 0.036	2.45 (1.12–5.36)
Milking method			*p* = 0.001	
Hand milking	98.5%	13.5		1.35 (0.85–2.14)
Machine milking	1.5%	10.0		Reference
Post-milking teat dipping			*p* < 0.05	
Practiced	20%	6.0		Reference
Not practiced	60%	16.0		2.67 (1.23–5.80)
Gloves used during milking			*p* < 0.05	
Yes	4.4%	5.0		Reference
No	95.6%	14.5		2.90 (1.05–8.02)
Parturition management			*p* < 0.05	
Separate calving pen	50%	8.7		Reference
Communal parturition	50%	20.9		2.40 (1.08–5.33)
Purpose of rearing (dual vs. dairy)	—	—	*p* = 0.688	NS
Water source (tube well vs. well)	3.7% tube	40.0	*p* = 0.095	NS

aOR = adjusted odds ratio from binary logistic regression (forward stepwise, Wald). NS = not significant. 95% CI = 95% confidence interval.

### 3.5 Clinical manifestations and impact on Milk production

All 20 CMT-positive animals exhibited udder swelling; 50% (*n* = 10) demonstrated palpable udder warmth. Milk colour changes to yellow or off-white were recorded in 85% (*n* = 17) of infected samples. Clot formation was observed in 60% (*n* = 12), and haematic discolouration occurred in 20% (*n* = 4). Ninety percent (18/20) of infected cows demonstrated measurable milk-yield reduction, and 50% (10/20) ceased production from at least one quarter. Full details are presented in Table 7.

**Table 7:** Clinical manifestations and milk quality changes in CMT-positive bovine mastitis cases, Jaffna District, 2024 (n = 20).

Clinical sign/milk change	Positive cases (*n*)	Prevalence (%)	Associated pathogen
Udder swelling	20	100.0	All
Udder warmth/pain on palpation	10	50.0	*S. aureus*
Milk colour change (yellow/off-white)	17	85.0	*S. aureus*, Strep.
Clot/flake formation in milk	12	60.0	*S. aureus*
Haematic discolouration (blood in milk)	4	20.0	*S. aureus*
Reduced milk yield	18	90.0	All
Complete cessation of milk (≥1 quarter)	10	50.0	*S. aureus* chronic

## 4 Discussion

This study provides the first district-wide characterisation of bovine mastitis prevalence, bacterial aetiology, antibiotic susceptibility, and farm-level risk factors in the Jaffna district of Northern Sri Lanka, addressing a long-standing evidence gap for this post-conflict region (7).

### 4.1 Prevalence and disease burden

The CMT-based point prevalence of 10.0% sits within the 8–15% range reported in comparable tropical smallholder systems (15, 16), but the much higher farmer-reported historical prevalence (26.5%) indicates that point-prevalence screening alone substantially underestimates the true herd-level burden of subclinical infection (3, 18). The complete absence of CMT-positive bulk-tank samples from Yarlco collection centres likely reflects dilution and quality-control screening at collection points (17) rather than a true absence of subclinical infection at farm level, and should not be interpreted as evidence of low community-level prevalence.

### 4.2 Bacterial aetiology

The dominance of *S. aureus* (50%) is consistent with reports from comparable South and Southeast Asian smallholder dairy systems, where staphylococci account for 30–60% of mastitis isolates (15, 19). *S. aureus* possesses a diverse array of virulence factors haemolysins, leukotoxins, protein A, biofilm formation, and beta-lactamase production — that collectively facilitate chronic, recurrent intramammary infection and impede antibiotic penetration into mammary parenchyma (20). The predominance of *S. aureus* in this district is plausibly linked to the high prevalence of hand milking (98.5%) and the 60% non-compliance with post-milking teat dipping, both recognised as primary transmission vectors for this contagious pathogen (21).

*Streptococcus agalactiae* (30%) is an obligate contagious pathogen transmitted cow-to-cow during milking via contaminated equipment and hands; its substantial prevalence here is consistent with shared milking equipment, limited sterilisation (only 72% of farmers used hot water), and the absence of segregation between CMT-positive and CMT-negative animals. Notably, *S. agalactiae* responds well to penicillin and cephalosporins when detected early, and systematic CMT screening combined with dry-cow therapy has achieved herd-level eradication in other resource-limited settings (22, 23). *E. coli and Klebsiella* spp. (10% each) are classical environmental pathogens, consistent with the predominantly well-water-based farm water supply (86.8%) and free-grazing practices that increase environmental bacterial exposure at the teat canal (24, 25).

### 4.3 Antibiotic susceptibility and implications for treatment

Enrofloxacin (90% susceptibility; MAR = 0.14) was the most effective antibiotic across all four pathogen groups, consistent with reports from Pakistan (15), Bangladesh (26), and Ethiopia (19), where fluoroquinolones have consistently shown the highest *in vitro* efficacy against bovine mastitis pathogens. Fluoroquinolones act by inhibiting bacterial DNA gyrase and topoisomerase IV, disrupting DNA replication and repair (27); their high oral bioavailability, tissue penetration, and accumulation in mammary tissue under acidic pH make them pharmacokinetically well suited to intramammary infection — consistent with the large mean inhibition zones recorded here (28.4 ± 6.2 mm).

By contrast, Oxytetracycline showed only 15% susceptibility (85% resistance) despite belonging to the same broad antibiotic class as Doxycycline (60% susceptibility), an eight-fold efficacy gap between two tetracycline-class agents. Doxycycline’s superior performance is attributable to its enhanced lipophilicity and tissue penetration relative to earlier tetracyclines, including documented activity against biofilm-forming *S. aureus* (28). The very low Oxytetracycline susceptibility most plausibly reflects the selective pressure of decades of widespread, largely unrestricted Oxytetracycline use in Sri Lankan smallholder livestock farming, a pattern documented across South and Southeast Asia (4), and the comparatively high MAR index for Oxytetracycline (0.45) is consistent with this interpretation. On this basis, Oxytetracycline should not be used as empirical first-line mastitis therapy in this district pending confirmatory culture and sensitivity testing.

Ciprofloxacin (55%) and Cephalexin (50%) showed moderate efficacy. Ciprofloxacin’s lower activity relative to Enrofloxacin is consistent with differences in CLSI breakpoints and the greater susceptibility of some mastitis pathogens to efflux-pump-mediated resistance against this agent (27). Cephalexin, a first-generation cephalosporin, showed the expected Gram-positive bias 6/10 *S. aureus* (60%) and 3/6 *S. agalactiae* (50%) isolates were susceptible, while both Gram-negative isolates were resistant, consistent with the limited Gram-negative spectrum of first-generation cephalosporins (9).

The MAR index findings warrant cautious interpretation given the small sample size (*n* = 20 isolates total, with only two *E. coli* and two *Klebsiella* isolates). An MAR index > 0.2 conventionally interpreted as indicating isolates from high-antibiotic-pressure environments (14) was observed for Doxycycline (0.22) through Oxytetracycline (0.45). This is broadly consistent with the questionnaire finding that 20% of farmers reported self-treating animals without veterinary guidance, although a causal link cannot be established from this cross-sectional design. The pattern of MAR indices approaching 0.5 for the least-effective antibiotics nonetheless suggests that multidrug-resistant isolates are present in a meaningful proportion of cases, a finding consistent with the broader regional concern outlined in the WHO Global Action Plan on AMR (29).

### 4.4 Farm-level risk factors

Semi-intensive farming systems showed the highest mastitis prevalence (14.5%; aOR 1.72; *p* = 0.05), representing 61% of surveyed farms. This is consistent with the hypothesis that shared cow sheds with restricted ventilation and imperfect bedding management favour survival and horizontal transmission of contagious pathogens particularly *S. aureus* and *S. agalactiae* between animals (24, 30), and is broadly in line with Haltia et al. (31), who reported elevated contagious-mastitis risk in semi-intensive Estonian herds relative to fully housed systems with automated milking hygiene controls.

European crossbred cattle (57% of the district population) showed the highest mastitis prevalence (14.6%; aOR 3.12; 95% CI 1.67–5.83; *p* < 0.05) relative to local breeds (5.2%; reference). This pattern is consistent with the literature suggesting comparatively lower innate udder immunity and higher milk yield-associated udder engorgement in high-yielding *Bos taurus*-type dairy breeds relative to locally adapted *Bos indicus*-derived breeds (11, 12). The independent association between daily milk yield > 10 L (aOR 2.45; *p* = 0.036) recorded in 99% of surveyed European crosses and mastitis occurrence may be mechanistically linked to elevated intramammary pressure and teat-sphincter patency during inter-milking intervals, both of which can facilitate pathogen entry (32); however, breed and yield are closely correlated in this dataset and their effects cannot be fully disentangled.

Non-compliance with post-milking teat dipping (60% of farmers; aOR 2.67; 95% CI 1.23–5.80; *p* < 0.05) and the near-universal absence of gloves during milking (95.6%; aOR 2.90; 95% CI 1.05–8.02; *p* < 0.05) were the two most modifiable, independently significant hygiene risk factors identified. Post-milking teat disinfection is one of the most consistently evidence-supported interventions for reducing contagious mastitis incidence, by eliminating residual bacteria on the teat surface before the sphincter closes after milking (21, 33); the low 20% compliance observed here likely reflects both limited farmer awareness and inconsistent availability of teat-dip products through the public veterinary network.

Communal parturition (50% of farms; aOR 2.40; *p* < 0.05) was associated with higher mastitis prevalence (20.9% versus 8.7% with separate calving pens). The periparturient period is widely regarded as a high-risk window for udder infection, owing to maximal teat-canal patency, transient suppression of neutrophil function, and environmental contamination from colostrum and uterine discharge (34). The provision of simple, low-cost calving-pen partitions — already practiced by half of the surveyed farms — represents a feasible, scalable intervention for this district.

### 4.5 Clinical and production impacts

The high proportion of CMT-positive cows showing reduced milk yield (90%) and complete cessation of production from at least one quarter (50%) is broadly consistent with reported production losses of 10–15% per cow per lactation for subclinical mastitis, rising to 25–50% in clinical cases (35). Altered milk composition associated with mastitis — increased SCC, elevated sodium and chloride, and reduced casein and lactose (36) — may also render affected milk less suitable for value-added processing into curd, ghee, or paneer, representing an additional, currently unquantified income loss for affected households beyond the direct yield reduction documented here.

### 4.6 Limitations

This study has several limitations that should be considered when interpreting the findings. First, the cross-sectional design captures point prevalence over a single six-month period and cannot establish causal relationships between risk factors and mastitis occurrence, nor capture seasonal variation in disease incidence. Second, the number of bacterial isolates available for antibiotic susceptibility testing was small (*n* = 20 overall, with only two isolates each of *E. coli* and *Klebsiella* spp.), so susceptibility estimates for these Gram-negative pathogens in particular should be regarded as preliminary. Third, species-level identification relied on phenotypic culture and biochemical methods rather than molecular confirmation (e.g., 16S rRNA sequencing or species-specific PCR), which may be less discriminatory for closely related taxa. Fourth, CMT is a screening test for elevated somatic cell count and may misclassify a small proportion of samples relative to direct SCC measurement or bacteriological culture of CMT-negative quarters. Finally, the MAR index findings, while indicative, are based on a small isolate panel and should be interpreted as hypothesis-generating rather than definitive evidence of region-wide antibiotic-pressure patterns.

## 5 Conclusion

This study provides the first district-wide characterisation of bovine mastitis prevalence, bacterial aetiology, antibiotic susceptibility, and farm-level risk factors in the Jaffna district of Northern Sri Lanka. A CMT-based point prevalence of 10.0% was established, alongside a higher historical farm-level burden of 26.5%. *Staphylococcus aureus* (50%) was the dominant causative pathogen, followed by *Streptococcus agalactiae* (30%), *E. coli* (10%), and *Klebsiella* spp. (10%). Enrofloxacin (90% susceptibility; MAR 0.14) and Doxycycline (60%) appear to be the most promising candidates for first- and second-line empirical treatment, while the 85% resistance to Oxytetracycline suggests this agent should be avoided for empirical therapy pending confirmatory susceptibility testing.

Logistic regression identified non-compliance with post-milking teat dipping (aOR 2.67), absence of gloves during milking (aOR 2.90), European crossbred cattle (aOR 3.12), communal parturition (aOR 2.40), and semi-intensive farming systems (aOR 1.72) as independent, modifiable factors associated with mastitis occurrence. These findings point toward a practical intervention agenda: (i) improved access to teat-dip solutions and milking gloves through the veterinary network; (ii) farmer training on milking hygiene and aseptic technique; (iii) low-cost calving-pen infrastructure; and (iv) establishment of a continuous antibiogram surveillance programme at the Jaffna VIC to monitor resistance trends over time.

Future research should incorporate automated SCC measurement, molecular confirmation of pathogen identity, *S. aureus* virulence-gene profiling and MRSA screening by mecA PCR, minimum inhibitory concentration (MIC) determination, seasonal incidence tracking, and longitudinal intervention studies to quantify the impact of targeted hygiene programmes on mastitis prevalence and antimicrobial resistance in the Jaffna district.

## Data Availability

The raw data supporting the conclusions of this article will be made available by the authors, without undue reservation.
